# Treatment of Mandibular Impacted Canine in a Patient with Class II Division 1 Malocclusion with “Reverse Pin”: A Case Report

**DOI:** 10.3390/medicina59101774

**Published:** 2023-10-05

**Authors:** Domenico Ciavarella, Marta Maci, Carlotta Fanelli, Mauro Lorusso, Michele Laurenziello, Lorenzo Lo Muzio, Marino Caroprese, Angela Pia Cazzolla, Michele Tepedino

**Affiliations:** 1 Department of Clinical and Experimental Medicine, University of Foggia, 71122 Foggia, Italy; 2 Department of Biotechnological and Applied Clinical Sciences, University of L’Aquila, 67100 L’Aquila, Italy

**Keywords:** impacted canine, class II malocclusion, canine surgery

## Abstract

This case report presents an orthodontic treatment conducted on a 13-year-old girl with bilateral Class II malocclusion and a mandibular impacted canine. The presence of an impacted tooth necessitates careful consideration of the timing of orthodontic treatment, the appropriate surgical procedure to expose the tooth, the specific orthodontic mechanics involved, and the potential problems that may arise, all of which depend on the type and location of the canine impaction in the jaw. The treatment plan included a surgical procedure to expose the impacted tooth and orthodontic traction to guide it into position. Correction of the Class II Division 1 malocclusion utilized a specialized technique called the “reverse pin”, reducing vertical side effects. The revised version maintains clarity and key information about the case report and treatment.

## 1. Introduction

When a permanent tooth fails to erupt within one year after its physiological period, it is considered impacted [1]. The following are the most commonly impacted teeth: mandibular wisdom teeth [2,3], maxillary canines [4,5,6], mandibular premolars [7], and maxillary central incisors [8]. Some authors report that mandibular canines are less frequently impacted compared to the maxillary ones [9].

A challenging clinical situation is when the crown of the impacted canine transmigrates over the midline with more than 50% of its length [10,11]. This occurrence is more unilateral than bilateral, with no specific gender differences and an incidence reported to range between 0.92% and 5.1%, while transmigration occurs with an incidence ranging from 1 to 3 [12]. 

The possible therapeutic treatments are:

- Extraction of the impacted tooth is performed in most situations [13];
- Surgical exposure followed by orthodontic repositioning [14,15];
- Autotransplantation [13].

Early diagnosis and treatment are essential for managing eruption anomalies and preventing close tooth anomalies [16]. 

The suitable orthodontic and surgical strategies will be determined based on the position of the canine in the jaw relative to adjacent teeth and bony cortices [9,17].

### Etiology

The leading causes of mandibular canine impaction are as follows:

- Unusual movement of the dental lamina during embryonic life;
- Inheritance factors;
- Cancers, endocrine gland dysfunction;
- Positive torque of lower incisors;
- When trauma at an early age closes the area of lower permanent canine eruption;
- Mechanical obstacles during the eruptive path of the tooth [13,18].

There are usually no clinical symptoms or signs except for the missed eruption of the canine beyond the chronological age of eruption. In such cases, a panoramic radiograph should be taken to identify the exact position of the canine [19].

Before initiating treatment, factors such as resorption of neighboring teeth, ankylosis, and coronal resorption of the included canine must be considered [19].

Although impaction of mandibular canines is less common than that of maxillary canines, therapeutic strategies appear to be similar [19]. Several authors have proposed hybrid therapeutic approaches using aligners, temporary anchorage devices (TADs), and sectionals shaped to promote tooth migration in the occlusal direction [20]. Other authors have suggested alternative treatment approaches; tooth position and intervention timing influence therapy choice [13]. The best therapy is the right one for the specific case; therefore, a correct diagnosis and a study of all records are the correct strategies for planning the most appropriate therapy [21].

This case report illustrates the successful treatment of an impacted lower canine in a buccal position, utilizing surgical exposure and orthodontic traction. Additionally, it combined with the correction of a class II Division I malocclusion using “reverse pin,” a modification of the posted system.

This clinical case’s distinctive feature lies in the treatment’s biomechanics. Thanks to meticulous anchorage management, an impacted tooth was successfully recovered without the need for orthodontic miniscrews. Moreover, class correction was achieved by leveraging the beneficial effects of elastics while minimizing undesired side effects.

## 2. Presentation of Case Report

A 13-year-old girl was presented to our office for evaluation. She had no history of dental trauma.

**Figure 1:**
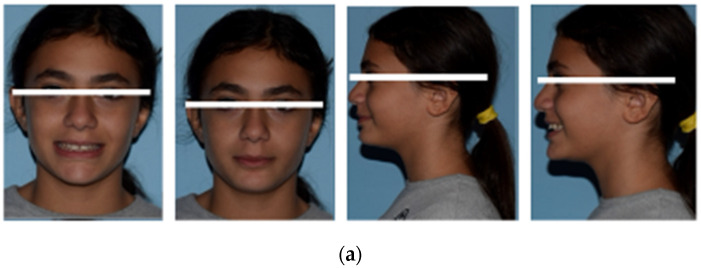

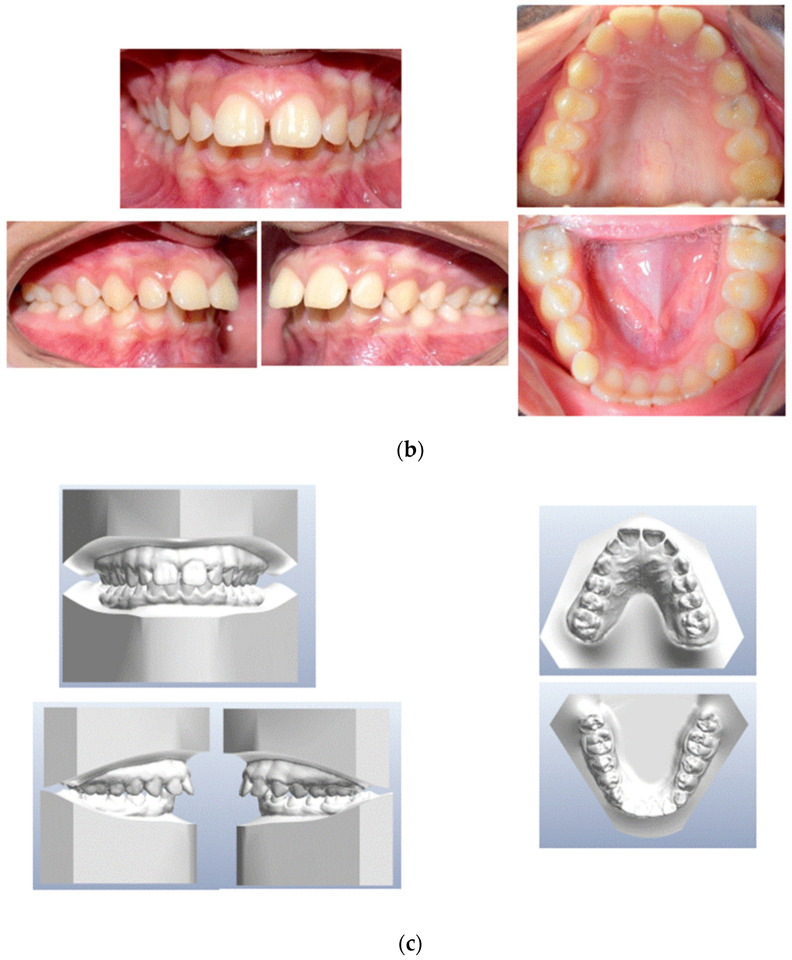
(**a**) Initial extraoral frontal smile–extraoral frontal rest and initial extraoral right profile photographs. (**b**) Pretreatment intraoral upper and lower occlusal image, right and left lateral photographs. (**c**) Pretreatment study models.

The pretreatment facial photographs showed a short facial pattern with reduced lower facial height, a symmetrical face, a slightly convex facial profile caused by maxillary prognathism, and a protrusive upper lip (Figure 1a).

The pretreatment intraoral photographs and dental casts showed a narrow arch form in both the upper and lower arches, along with a Class II canine and molar relationship on both sides. The patient had a mild overbite and a large overjet, exhibited lip competence, and reported no functional problems. Oral hygiene was good, with no evidence of decay or periodontal inflammation. Additionally, there was a retained deciduous canine in the lower arch, well-aligned upper and lower midlines, and unerupted third molars (Figure 1b,c).

**Figure 2:**
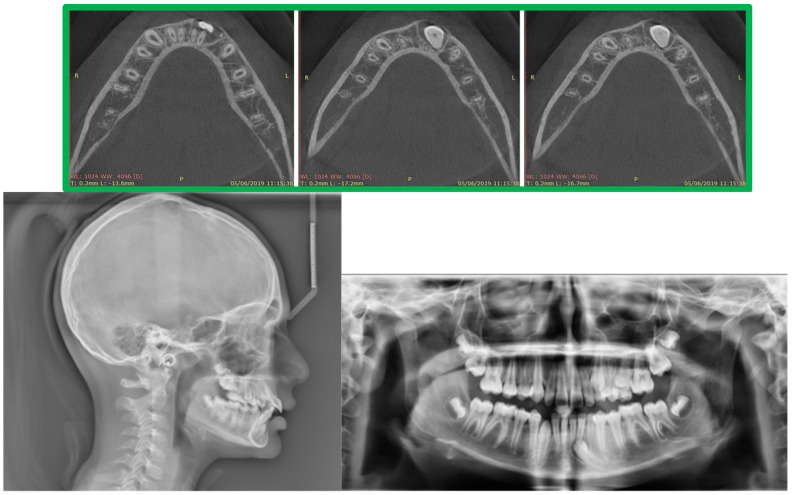
CBCT, cephalometric radiographs, and pretreatment panoramic.

The panoramic radiograph and CBCT revealed a horizontally impacted canine in a buccal position below the root apex of the inferior incisors, completely covered by bone (Figure 2). The vitality test was performed, and the lower left incisor was found to be vital. Computerized axial tomography was used to plan the surgical exposure of the canine for orthodontic engagement and traction.

**Figure 3:**
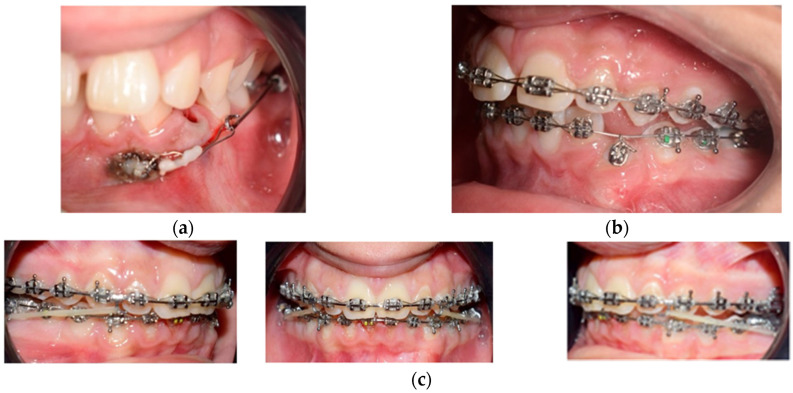
(**a**,**b**) The mandibular left canine was surgically exposed by using a laser. A bottom was placed labially near the tip of the cusp combined with a ligature without being sutured. (**c**) Intraoral views of treatment: 0.019 × 0.025 stainless steel wire upper and lower was inserted. The reverse pin to start class II biomechanics was placed on 13 and 23, with class II elastics (upper canines and first inferior molars) weighing 3/8 4.5 ounces.

Cephalometric tracings (Table 1) showed a skeletal Class II relationship (ANB angle 4.2°) with reduced divergence angle (Sn-GoMe 22.2°), maxillary prognathism (SNA angle 85.2°), and a well-positioned jaw (SNB angle 81.3°); the upper incisors (+1 to SN 116.15°) and lower incisors are proclined (−1 to GoMe 109.72°) with a reduced interincisal angle (111.98°) (Figure 3). The other cephalometric values are presented in Table 1.

After clinical and radiological evaluations, the patient was diagnosed with a Class II Division 1 malocclusion, characterized by brachyfacial features, maxillary protrusion, a deep bite, large overjet, proclined incisors, several impacted lower left canines, and retained wisdom teeth. The patient's request was to reduce the overjet and orthodontically correct the impacted mandibular canine.

### 2.1. Treatment Objectives

- Improve overjet and overbite;
- Achieve bilateral canine and molar class I occlusion;
- Increase facial esthetic balance;
- Level the arches and make both coordinate with each other;
- Orthodontic correction of the impacted lower left canine.

### 2.2. Treatment Alternatives

- Surgical removal of the impacted tooth and conventional bridge or a fixed prosthesis on implant rehabilitation;
- Auto-transplantation, possibly followed by endodontic treatment of mandibular left canine;
- Surgical exposure of impacted left canine following orthodontic alignment in dental arch [22].

In some patients where the above-mentioned alternatives are not feasible, another option could be to leave the impacted canine untreated. In such instances, frequent radiographic monitoring is crucial to assess the situation [23].

To treat the Class II malocclusion in this patient, the authors decided against using a functional appliance like extra-oral traction to control maxillary prognathism due to the patient’s maturity level (CS5 stage, determined using the cervical vertebral maturation method) [24]. An alternative treatment could involve extracting upper premolars and employing the traditional multibrackets technique for distalization [25]. 

In the present case, a non-extraction orthodontic treatment combined with class II elastic was performed, considering the patient's slight skeletal discrepancy. 

### 2.3. Treatment Progress

The treatment sequence adopted is presented below:

- Surgical exposure of the left mandibular canine using a diode laser (Doctor Smile, Lambda Spa, Brendola, Vi, Italy) for operculectomy and excision of fibromucous tissue. The corticotomy was performed using an ultrasonic piezosurgery system (Mectron Medical Technology, Carasco, Genova, Italy). A bottom (3M Unitek, Monrovia, CA, USA) was placed labially as close as possible to the canine’s coronal tip, combined with a metallic ligature and a cantilever (0.016 × 0.022 TMA, American Orthodontics, Sheboygan, WI, USA) without sutures (Figure 3a,b). The cantilever allowed for distal and buccal traction with light forces (1.7–2.8 ounces). A lingual arch was soldered (passive only on 31–32);
- Fixed multibrackets appliance (American Orthodontics Corp., Sheboygan, WI, USA, MBT prescription) were placed in the upper arch with 0.014 NiTi wire, bent back for controlling anchorage;
- After one month, a 0.016 NiTi wire was inserted in the upper arch;
- Fixed multibracket appliances were placed in the lower arch with a 0.014 NiTi wire, starting vertical traction of the canine. A bent back was used for controlling anchorage;
- After two months, the inferior left canine achieved a vertical position and reached its position in the arch. The bracket was replaced to adjust its axial inclination, and then a 0.016 NiTi wire was inserted;
- Before using stainless steel wire, a ligature from 16 to 26 and 36 to 46 for controlling anchorage was placed;
- 0.016 × 0.022 stainless steel wire was inserted in the upper and lower arches;
- 0.019 × 0.025 stainless steel wire was inserted in the upper and lower arches;
- Reverse Pin System” was placed on 13 and 23 to initiate class II biomechanics. Class II elastics (upper canines and first inferior molars) 3/8 4.5 ounces were used (Figure 3c). Class II elastics were used for 18 h every day;
- One year later, class I occlusion was achieved, and the class II elastics were used only during nighttime;
- Two years later, the appliance was deboned, and positioner retainers were given to the patient with the prescription to wear them every night.

**Figure 4:**
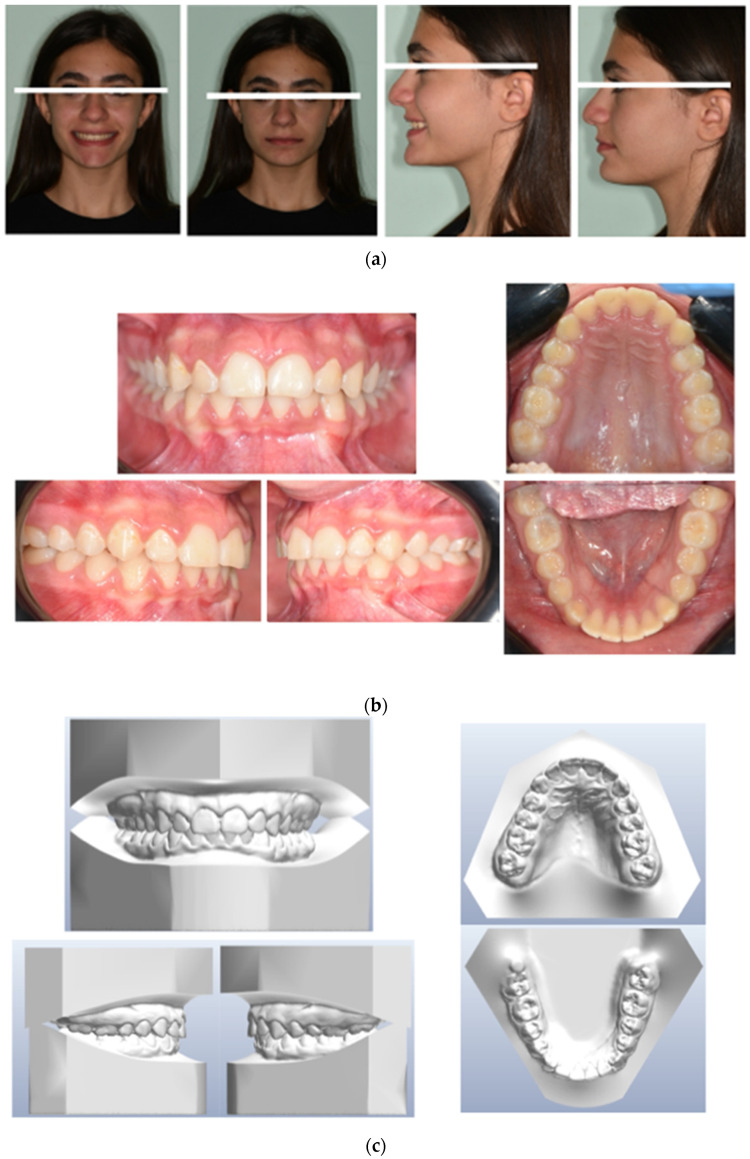
(**a**) Post-treatment extraoral frontal smile–extraoral frontal rest and extraoral right profile photographs. (**b**) Post-treatment intraoral upper and lower occlusal image, frontal, right, and left lateral photographs. (**c**) Final study models.

**Figure 5:**
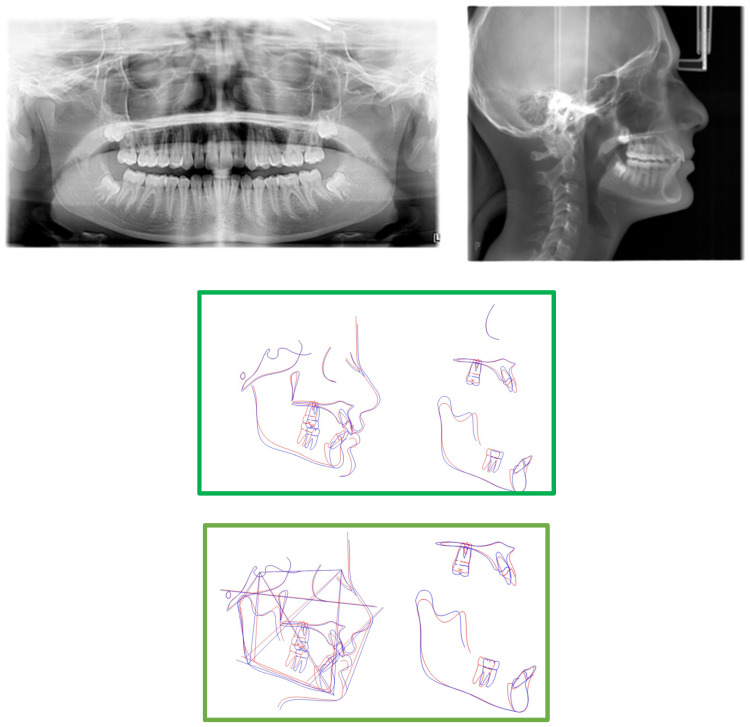
Post-treatment panoramic, cephalometric radiographs, tracing, and superimpositions. (Pre-treatment is is portrayed in blue, post-tretment is portrayed in red).

**Figure 6:**
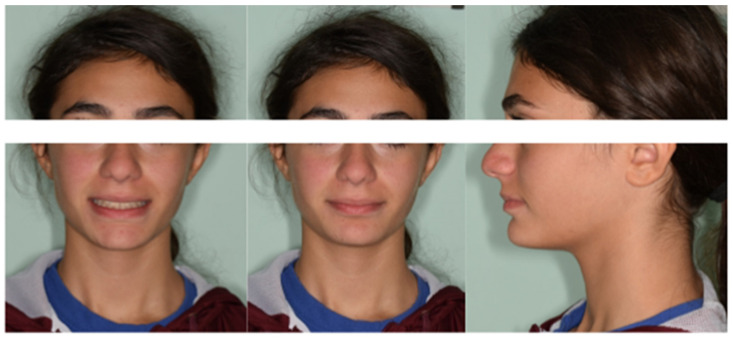

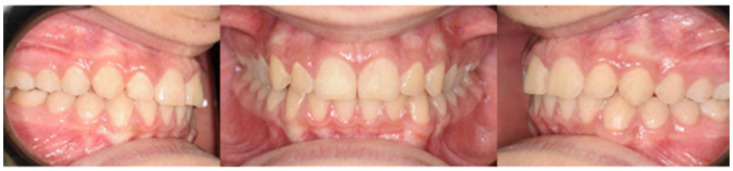
The 5-year follow-up shows the stability of the results obtained.

After 2 years of treatment, significant improvements were achieved in the aesthetic profile (Figure 4a) and occlusal relationship. Class I molar and canine relationships, along with good overjet and overbite, were achieved after treatment (Figure 4b,c). In the final panoramic radiograph, there was no evidence of root or alveolar bone resorption (Figure 5). Cephalometric analysis (Table 1) revealed no significant change in SNA–SNB, an improvement in ANB, and the position of the incisors changed from 116.15° to 113.37° in relation to SN line, and the position of the mandibular incisor changed from 109.72° to 114.5° in relation to GoMe line. Maxillary incisor proclination decreased by 3°, while mandibular incisors improved by 5° (Table 1). Superimposition showed an improvement of overjet and overbite and front rotation of the occlusal plane due to the use of reverse pin vertical control (Figure 5). Dental casts and intraoral photographs after treatment exhibited a well-exposed lower left canine (Figure 4b,c). The stability of the results was confirmed by a 5-year follow-up assessment (Figure 6). 

## 3. Discussion

Impacted teeth require a multidisciplinary approach involving surgeons and orthodontists. This case report discussed two orthodontic topics: mandibular canine impaction and treatment of class II malocclusion in an adult patient. 

Based on the scientific literature [26,27], when there is insufficient space for mandibular canine eruption, surgical extraction is the most commonly performed treatment [22]. In this case, we aimed to preserve the deciduous canine as long as possible by restoring it and simulating a permanent canine with a composite buildup. However, in agreement with D’Alessandri et al., long-term preservation of the deciduous tooth may not be realistic, and the patient may eventually require prosthodontic or implant-supported rehabilitation [13]. 

Autotransplantation is not commonly used due to a lack of data from long-term studies. According to Machado et al. [28], the 6-year survival rates for canines with complete root formation ranged from 75.3% to 91%, but several conditions should be considered, such as signs of ankylosis or root reabsorption [29].

Undoubtedly, orthodontic traction is a complex strategy, but it proves to be the most efficient therapy for achieving a correct dentoalveolar relationship. In our case, maintaining proper anchorage control was crucial, utilizing a lingual arch and lace backs without the need for mini screws [26]. Although miniscrews have several advantages in anchorage management, special attention has been focused on preserving periodontal health [30,31]. An accurate preliminary CBCT was necessary to identify the position of the impacted canine and determine the appropriate treatment approach. If the canine is in a favorable position, not deeply embedded in the alveolar bone, orthodontic traction should be the preferred option due to its long-term stability of results [32]. 

In this patient, the recovery of the canine occurred with distal and buccal traction using an adequate cantilever, which allowed eruption into the attached gingiva and prevented damage to the roots of the lateral incisors. The canine was able to “circumnavigate” the root of the lateral incisors without compromising the buccal bone cortex. The use of a statically determined light force system made it possible to reduce the risk of complications while respecting bone biology.

Another important benefit of the orthodontic treatment performed was the maintenance of periodontal health and achievement of functional occlusal rehabilitation. By preserving the patient’s teeth and their anatomy, good canine guidance during excursive movements was obtained, and the gingival parabolas of the canines reached the same level as the central incisors, improving the aesthetics of the smile [33]. 

**Figure 7:**
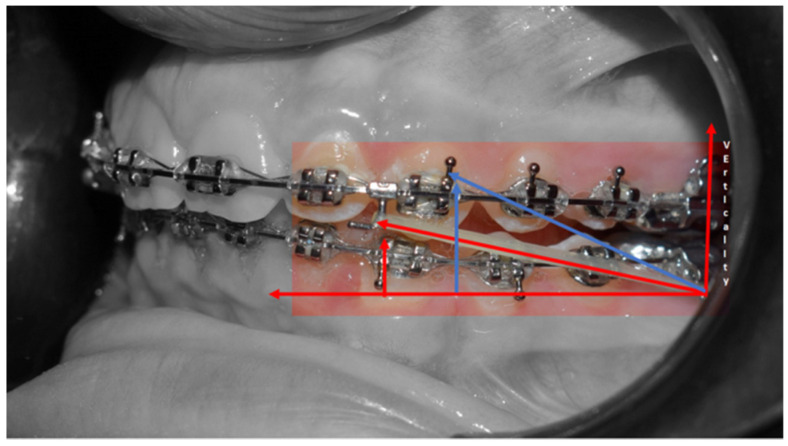
Reverse pin system.

Janson et al. suggest that correcting class II malocclusion in an adult patient is possible with class II elastics, upper molar distalization, upper premolar extraction, and frontal teeth retraction using the multibrackets technique [34]. Treating class II malocclusion in adults without extractions can be challenging, requiring a well-personalized treatment plan with careful control of orthodontic mechanics to minimize side effects. In this patient, the superior part of the face was slightly protruded, while the lower jaw was well balanced. Class II elastics to bring the mandibular dentition forward were the preferred therapy [35]. However, according to Janson et al. [34], class II elastics may produce vertical and sagittal effects, such as extrusion of upper incisors, proclination of inferior incisors, loss of mandibular anchorage, and if we did not desire a mandibular post-rotation, are not indicated. To avoid mandibular post-rotation, the “Reverse Pin System” (Figure 7) was utilized. In this system, class II elastics were applied to the clamped pin in an occlusal direction to distance the application of force from the center of resistance of the lower molars and upper incisors, decreasing the side effects of extrusive forces [36]. This system optimizes the effect of class II elastics for correcting the molar and canine class while decreasing the vertical component of the elastic traction and, consequently, the post-rotation of the occlusal plane.

Cephalometric superimposition demonstrated the effects of the class II elastics on the reverse pin, showing significant mandibular dentition advancement and vertical control of the occlusal/mandibular plane (Figure 5).

## 4. Conclusions

Orthodontic traction and restoration of impacted canines in the arches should be considered as an effective alternative treatment. The use of class II elastics with the reverse pin in adult patients allows for dentoalveolar correction of the occlusion without vertical side effects. The treatment time is lengthy, requiring patient compliance, but the results demonstrate good efficacy, stability of the occlusion, and good aesthetics.

## Figures and Tables

**Table 1:** Initial and final cephalometric analysis (Jarabak).

Measurement	Initial	Final	Norm
S^N^A	85.2°	85.5°	81°
S^N^B	81.3°	81.8°	79°
A^N^B	4.2°	3.7	2°
SN^Pog	82.5°	84.3°	80°
S	107.7°	122.8°	123° ± 5°
AR	168.2°	143°	143° ± 6°
Go	106.2	111.4°	130° ± 7°
Upper gonial angle	43.2°	50°	52°–55°
Lower gonial angle	63°	61.3°	70°–75°
N^S^Ar^Go^Gn	382.1°	377.2°	396°
Sn/Go-Me	22.2°	17.1°	32°
SN	67.6 mm	69.7 mm	71 mm
GoMe	72.3 mm	78.3 mm	71 mm
S-Ar	41.3 mm	32.6 mm	32 mm
Ar-Go	33.1 mm	53.8 mm	44 mm
Dentoalveolar component			
UI^SN	116.15°	113.37°	102° ± 2°
LI^GOME	109.72°	114.5°	90° ± 3°
Interincisal angle	111.98°	115.07°	135°

## Data Availability

The data presented in this study are available on request from the corresponding author.

## References

[1] Koc A., Kaya S., Abdulsalam W.A. (2021). Three-Dimensional Analysis of Impacted Maxillary and Mandibular Canines and Evaluation of Factors Associated with Transmigration on Cone-Beam Computed Tomography Images. J. Oral Maxillofac. Surg. Off. J. Am. Assoc. Oral Maxillofac. Surg..

[2] Santosh P. (2015). Impacted mandibular third molars: Review of literature and a proposal of a combined clinical and radiological classification. Ann. Med. Health Sci. Res..

[3] Crincoli V., Cazzolla A.P., Di Comite M., Muzio L.L., Ciavarella D., Dioguardi M., Bizzoca M.E., Palmieri G., Fontana A., Giustino A. (2021). Evaluation of Vitamin D (25OHD), Bone Alkaline Phosphatase (BALP), Serum Calcium, Serum Phosphorus, Ionized Calcium in Patients with Mandibular Third Molar Impaction. An Observational Study. Nutrients.

[4] Tepedino M., Laurenziello M., Guida L., Montaruli G., Grassia V., Chimenti C., Campanelli M., Ciavarella D. (2020). Sella turcica and craniofacial morphology in patients with palatally displaced canines: A retrospective study. Folia Morphol..

[5] Laurenziello M., Montaruli G., Gallo C., Tepedino M., Guida L., Perillo L., Ciavarella D. (2017). Determinants of maxillary canine impaction: Retrospective clinical and radiographic study. J. Clin. Exp. Dent..

[6] Crincoli V., Tettamanti L., Lucchina A.G., Dedola A., Cazzolla A.P., Lacaita M.G., Mastrangelo F. (2019). Correlation between maxillary canine impaction and facial biotype. J. Craniofacial Surg..

[7] Kalia V., Aneja M. (2009). Mandibular premolar impaction. Sch. Res. Exch..

[8] Jain S., Raza M., Sharma P., Kumar P. (2021). Unraveling impacted maxillary incisors: The why, when, and how. Int. J. Clin. Pediatr. Dent..

[9] Jain S., Debbarma S. (2019). Patterns and prevalence of canine anomalies in orthodontic patients. Med. Pharm. Rep..

[10] Azeem M., Afzal A., Ahmed Z., Ali M.M., Haq A.U., Hamid W.U. (2019). Investigation of transmigrated mandibular canines. Dent. Press J. Orthod..

[11] Mupparapu M. (2002). Patterns of intra-osseous transmigration and ectopic eruption of mandibular canines: Review of literature and report of nine additional cases. Dentomaxillofac Radiol..

[12] Alhammadi M.S., Asiri H.A., Almashraqi A.A. (2018). Incidence, severity and orthodontic treatment difficulty index of impacted canines in Saudi population. J. Clin. Exp. Dent..

[13] Dalessandri D., Parrini S., Rubiano R., Gallone D., Migliorati M. (2017). Impacted and transmigrant mandibular canines incidence, aetiology, and treatment: A systematic review. Eur. J. Orthod..

[14] Crescini A., Baccetti T., Rotundo R., Mancini E.A., Prato G.P. (2009). Tunnel technique for the treatment of impacted mandibular canines. Int. J. Periodontics Restor. Dent..

[15] Plaza S.P. (2016). Orthodontic traction of a transmigrated mandibular canine using mini-implant: A case report and review. J. Orthod..

[16] Cannavale R., Matarese G., Isola G., Grassia V., Perillo L. (2013). Early treatment of an ectopic premolar to prevent molar-premolar transposition. Am. J. Orthod. Dentofacial. Orthop..

[17] Aydin U., Yilmaz H.H., Yildirim D. (2004). Incidence of canine impaction and transmigration in a patient population. Dentomaxillofac Radiol..

[18] Bertl M.H., Frey C., Bertl K., Giannis K., Gahleitner A., Strbac G.D. (2018). Impacted and transmigrated mandibular canines: An analysis of 3D radiographic imaging data. Clin. Oral Investig..

[19] Grybiene V., Juozenaite D., Kubiliute K. (2019). Diagnostic methods and treatment strategies of impacted maxillary canines: A literature review. Stomatologija.

[20] Greco M., Machoy M. (2022). Impacted Canine Management Using Aligners Supported by Orthodontic Temporary Anchorage Devices. Int. J. Env. Res. Public Health.

[21] Karabas H.C., Ozcan I., Erturk A.F., Guray B., Unsal G., Senel S.N. (2021). Cone-beam computed tomography evaluation of impacted and transmigrated mandibular canines: A retrospective study. Oral Radiol..

[22] Stabryła J., Plakwicz P., Kukuła K., Zadurska M., Czochrowska E.M. (2021). Comparisons of different treatment methods and their outcomes for impacted maxillary and mandibular canines: A retrospective study. J. Am. Dent. Assoc..

[23] Herrera-Atoche J.R., Esparza-Villalpando V., Martínez-Aguilar V.M., Carrillo-Ávila B.A., Escoffié-Ramírez M. (2021). Treatment options for mandibular canine transmigration - a case series based on dental literature. Br. J. Oral Maxillofac. Surg..

[24] Männchen R., Serafin M., Fastuca R., Caprioglio A. (2022). Does Early Treatment Improve Clinical Outcome of Class II Patients? A Retrospective Study. Children.

[25] Proffit W.R., Fields H.W., Larson B., Sarver D.M. (2018). Contemporary Orthodontics—E-Book.

[26] Bertl M.H., Frey C., Bertl K., Giannis K., Gahleitner A., Strbac G.D. (2018). Reply to: Letter to the editor about the article published in Clinical Oral Investigations tilted: Impacted and transmigrated mandibular canines: An analysis of 3D radiographic imaging data (Bertl MH, Frey C, Bertl K, Giannis K, Gahleitner A, Strbac GD (2018) Clin Oral Investig. https://doi.org/10.1007/s00784-018-2342-0. Oral Investig..

[27] Díaz-Sánchez R.-M., Castillo-De-Oyagüe R., Serrera-Figallo M., Hita-Iglesias P., Gutiérrez-Pérez J.-L., Torres-Lagares D. (2016). Transmigration of mandibular cuspids: Review of published reports and description of nine new cases. Br. J. Oral Maxillofac. Surg..

[28] Machado L., Nascimento R.D., Ferreira D., Mattos C., Vilella O. (2016). Long-term prognosis of tooth autotransplantation: A systematic review and meta-analysis. Int. J. Oral Maxillofac. Surg..

[29] Grisar K., Nys M., The V., Vrielinck L., Schepers S., Jacobs R., Politis C. (2019). Long-term outcome of autogenously transplanted maxillary canines. Clin. Exp. Dent. Res..

[30] Moeini N., Sabri H., Galindo-Fernandez P., Mirmohamadsadeghi H., Valian N.K. (2023). Periodontal status following orthodontic mini-screw insertion: A prospective clinical split-mouth study. Clin. Exp. Dent. Res..

[31] Albogha M.H., Takahashi I. (2019). Effect of loaded orthodontic miniscrew implant on compressive stresses in adjacent periodontal ligament. Angle Orthod..

[32] Majumdar S.K., Hossain A., De N., Chadda D., Bachhar M.K., Mishra S. (2020). Effect of Diagnosis by Two-Dimensional Radiography Versus CBCT on Surgical Aspects of Transmigrated Impacted Mandibular Canines. J. Maxillofac. Oral Surg..

[33] Crescini A., Nieri M., Buti J., Baccetti T., Mauro S., Pini Prato G.P. (2007). Short- and long-term periodontal evaluation of impacted canines treated with a closed surgical-orthodontic approach. J. Clin. Periodontol..

[34] Janson G., Sathler R., Fernandes T.M.F., Branco N.C.C., de Freitas M.R. (2013). Correction of Class II malocclusion with Class II elastics: A systematic review. Am. J. Orthod. Dentofac. Orthop..

[35] Sambataro S., Bocchieri S., Bafumi L., Fiorillo L., Cervino G., Cicciù M. (2019). Elastics Selector Gauge as Orthodontics Device Applied to Inter-Maxillary Traction during Malocclusion Correction. J. Funct. Morphol. Kinesiol..

[36] Pontes L.F., Maia F.A., Almeida M.R., Flores-Mir C., Normando D. (2017). Mandibular Protraction Appliance Effects in Class II Malocclusion in Children, Adolescents and Young Adults. Braz. Dent. J..

